# Crystal structure and Hirshfeld surface analysis of tris­(acetohydrazide-κ^2^ *N*,*O*)(nitrato-κ*O*)(nitrato-κ^2^ *O*,*O*′)terbium(III) nitrate

**DOI:** 10.1107/S2056989022002298

**Published:** 2022-03-08

**Authors:** Chatphorn Theppitak, Sakchai Laksee, Kittipong Chainok

**Affiliations:** a Thammasat University Research Unit in Multifunctional Crystalline Materials and Applications (TU-McMa), Faculty of Science and Technology, Thammasat University, Khlong Luang, Pathum Thani, 12121, Thailand; b Nuclear Technology Research and Development Center, Thailand Institute of Nuclear Technology (Public Organization), Ongkharak, Nakon Nayok, 26120, Thailand

**Keywords:** crystal structure, acetohydrazide, Hirshfeld surface analysis, lanthanide(III) ion, terbium(III)

## Abstract

In the title lanthanide(III) compound, [Tb(NO_3_)_2_(C_2_H_6_N_2_O)_3_]NO_3_, the asym­metric unit contains one Tb^3+^ ion, three acetohydrazide (C_2_H_6_N_2_O) ligands, two coordinated nitrate anions, and an isolated nitrate anion. The Tb^3+^ ion is in a ninefold coordinated distorted tricapped trigonal–prismatic geometry formed by three oxygen atoms and three nitro­gen atoms from three different acetohydrazide ligands and three oxygen atoms from two nitrate anions. In the crystal, the complex mol­ecules and the non-coordinated nitrate anions are assembled into a three-dimensional supra­molecular architecture through extensive N—H⋯O hydrogen-bonding inter­actions between the amine NH groups of the acetohydrazide ligands and the nitrate oxygen atoms. Hirshfeld surface analysis was performed to aid in the visualization of inter­mol­ecular contacts.

## Abstract 2

The crystal structure and supra­molecular inter­actions of a new terbium(III) complex with an acetohydrazide ligand are reported.

## Chemical context

Over the past two decades, there has been increasing inter­est in the construction of new lanthanide-based coordination compounds, not only because of their structural diversity but also because of their fascinating potential applications in luminescence, magnetism, adsorption, and similar areas (Roy *et al.*, 2014 ▸; Cui *et al.*, 2018 ▸; Kuwamura *et al.*, 2021 ▸). It is well known that lanthanide(III) ions have a high affinity for and prefer binding to hard donor atoms. Thus, organic ligands with oxygen donor atoms such as aromatic polycarb­oxy­lic acids have been used extensively for the formation of these coordination materials (Janicki *et al.*, 2017 ▸) whereas organohydrazide ligands have received far less attention. Accordingly, a ConQuest search of the Cambridge Structural Database (CSD, Version 5.42, September 2021 update; Bruno *et al.*, 2002 ▸; Groom *et al.*, 2016 ▸) reveals only 23 entries for hydrazide-containing lanthanide complexes. Among them, 15 lanthanide coordination complexes have recently been reported by our groups. Some of these complexes exhibited a high CO_2_ uptake ability at high pressure (Theppitak *et al.*, 2021*a* ▸), and have shown great potential as luminescent sensors for acetone and the Co^2+^ ion with good recyclability (Theppitak *et al.*, 2021*b* ▸). In this work, we present the mol­ecular structure of a new terbium(III) complex, [Tb(C_2_H_6_N_2_O)_3_(NO_3_)_2_]NO_3_ (**1**), synthesized with acetohydrazide (C_2_H_6_N_2_O) as the organic ligand. In addition, a Hirshfeld surface analysis and two-dimensional fingerprint plots were used to qu­antify the inter­molecular contacts in the crystal structure. 
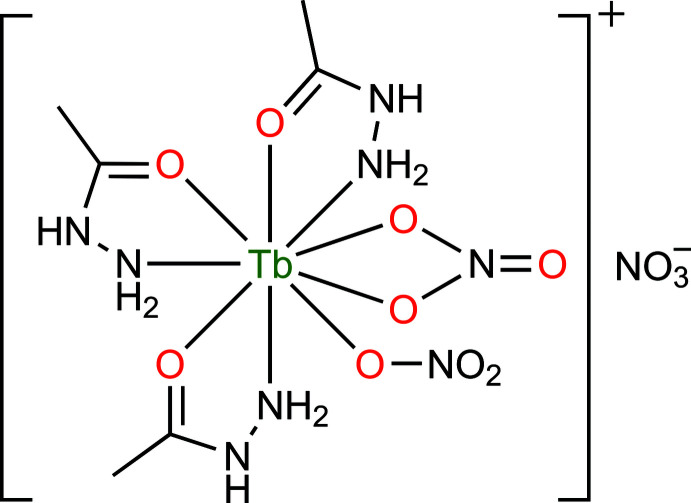


## Structural commentary

**Figure 1:**
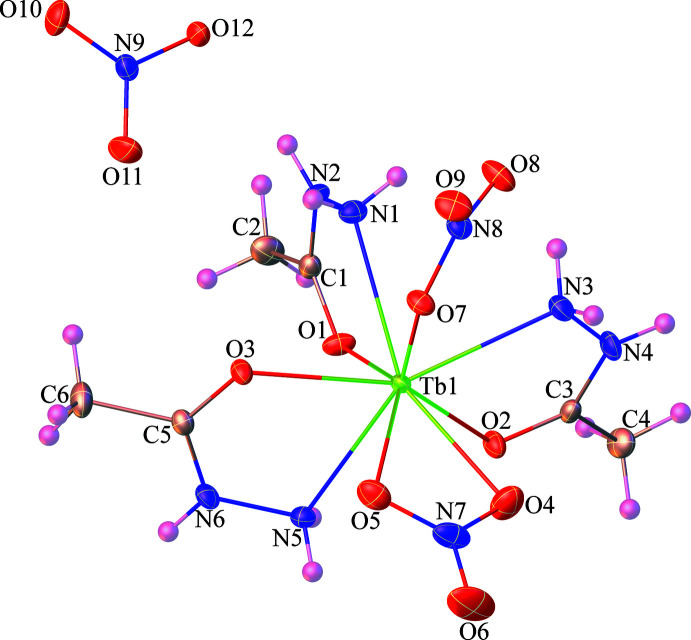
Mol­ecular structure of **1**, showing the atom-labeling scheme. Displacement ellipsoids are drawn at the 50% probability level.

**Figure 2:**
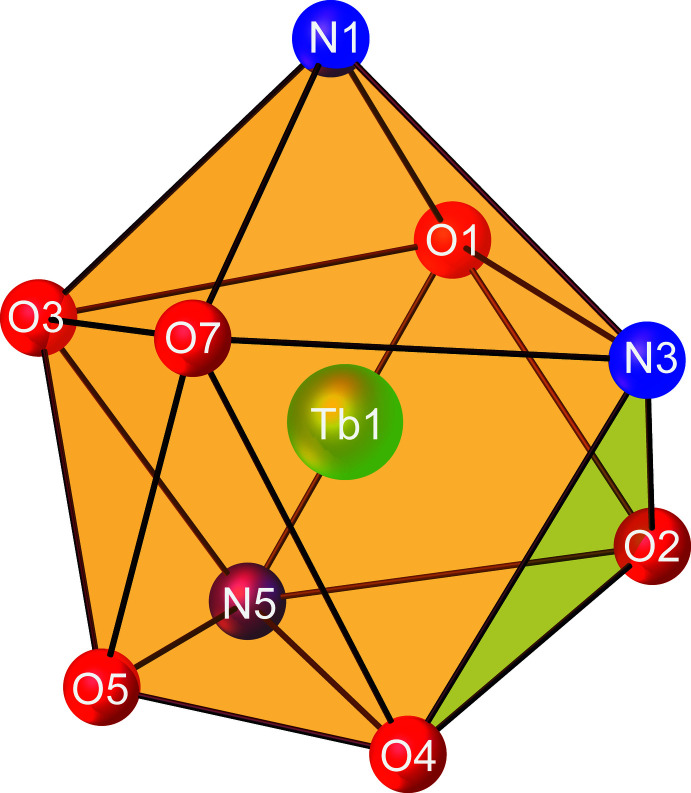
View of the distorted tricapped trigonal–prismatic coordination geometry of the central Tb^3+^ atom in **1**.

The mol­ecular structure of **1** is shown in Fig. 1 ▸. The asymmetric unit contains one Tb^3+^ ion, three acetohydrazide ligands, two coordinated nitrate anions, and a non-coordinated nitrate counter-anion. The Tb^3+^ ion is ninefold coordinated (TbN_3_O_6_) by three nitro­gen atoms and three oxygen atoms from three different acetohydrazide ligands, two oxygen atoms from one chelate nitrate anion, and one oxygen atom from another nitrate anion. As can be seen in Fig. 2 ▸, the coordination polyhedron of the Tb^3+^ ion is best described as having a distorted tricapped trigonal–prismatic geometry, wherein the N3, N5, O1, O3, O4, and O7 atoms form a trigonal prism, while the N1, O2, and O5 atoms act as caps. The Tb—O bond lengths of 2.353 (2)–2.496 (2) Å are slightly shorter than the Tb—N bond lengths [2.553 (2)–2.586 (2) Å]. The bond angles around the central Tb^3+^ ion fall into the range of 50.93 (7)–150.97 (7)°. These values are comparable to those reported for other ninefold-coordinated Tb^3+^ compounds containing oxygen/nitro­gen-donor ligands such as [Tb(C_17_H_13_N_3_)(NO_3_)_2_(DMSO)]·CH_3_OH (VUKNEW, Chen *et al.*, 2015 ▸) and [Tb(C_13_H_22_N_3_)(NO_3_)_3_]·MeCN (SEZTOJ, Long *et al.*, 2018 ▸).

## Supra­molecular features

**Figure 3:**
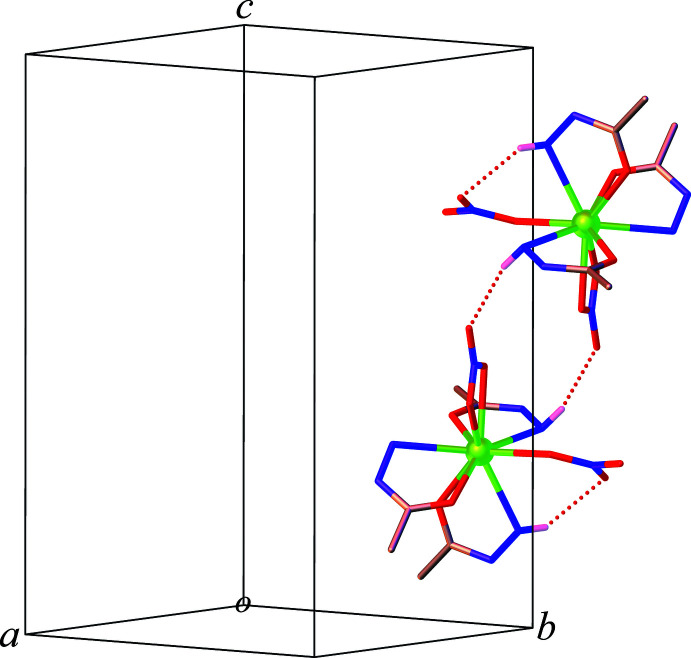
Dimer formation through N—H⋯O hydrogen bonds (dashed lines) in **1** (hydrogen atoms, except those forming hydrogen bonds, are omitted for clarity).

**Figure 4:**
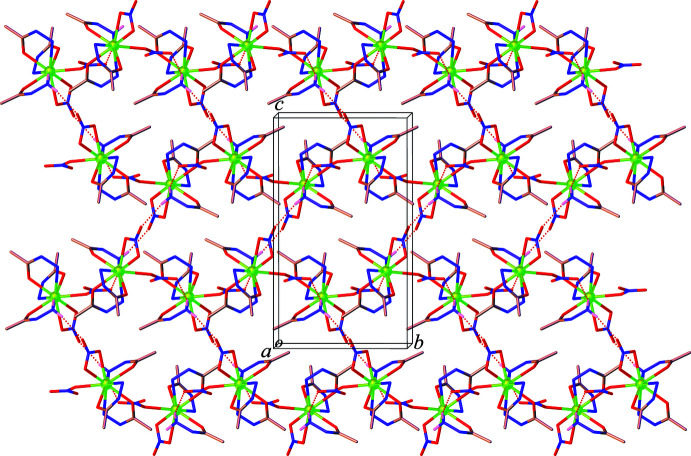
The two-dimensional hydrogen bonded layer in **1** (hydrogen atoms, except those forming hydrogen bonds, are omitted for clarity).

**Figure 5:**
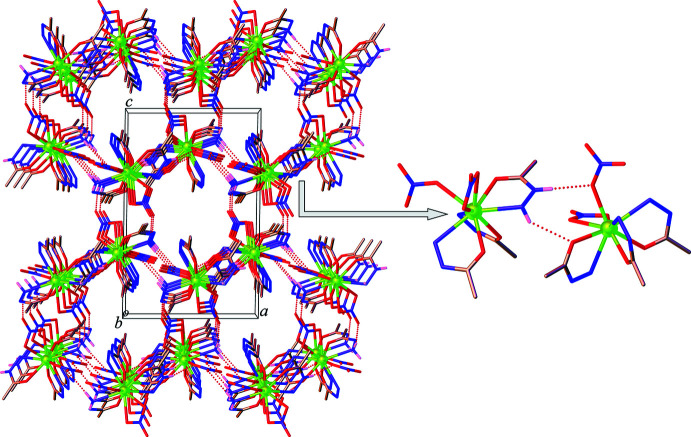
The three-dimensional hydrogen-bonded network in **1** (hydrogen atoms, except those forming hydrogen bonds, are omitted for clarity).

**Figure 6:**
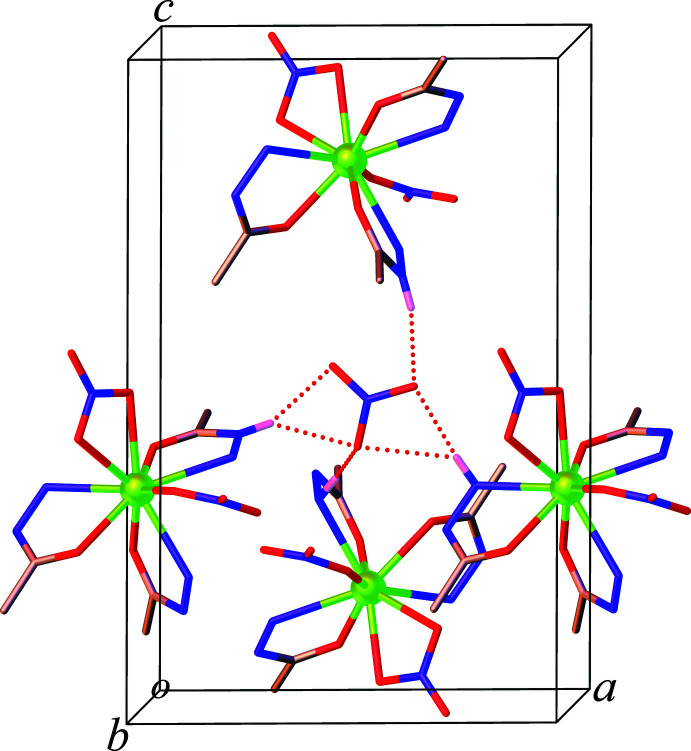
View of **1** approximately along the *b-*axis direction, showing the N—H⋯O hydrogen-bonding inter­actions involving the non-coordinated nitrate ion and the complex mol­ecules (hydrogen atoms, except those forming hydrogen bonds, are omitted for clarity).

Extensive hydrogen-bonding inter­actions involving the three components of the hydrazide group of the acetohydrazide ligand and the coordinated and non-coordinated nitrate ions contribute to the stabilization of the supra­molecular structure of **1** (Table 1 ▸; the N—H distances are all fixed with N—H = 0.86 ± 0.02 Å). A closer inspection of the structure reveals that the [Tb(C_2_H_6_N_2_O)_3_(NO_3_)_2_]^+^ complex mol­ecules form centrosymmetric dimers *via* pairs of symmetry-related N3—H3*B*⋯O6 hydrogen bonds involving the amine NH group of the acetohydrazide ligand and the coordinated nitrate oxygen atom, Fig. 3 ▸. Notably, the amine NH donor and the coordinated nitrate oxygen acceptor is also involved in an intra­molecular N1—H1*A*⋯O8 hydrogen bond. The dimers are further held together through an inter­molecular N3—H3*A*⋯O9 hydrogen bond between the amine NH and the coordinated nitrate oxygen (O9), resulting in the formation of a two-dimensional supra­molecular layer that propagates in the [100] direction, Fig. 4 ▸. Ultimately, adjacent layers are connected into a three-dimensional supra­molecular architecture *via* the other two complementary N—H⋯O hydrogen-bonding inter­actions (*i.e.* N5—H5*B*⋯O3 and N6—H6⋯O7) occurring between the acetohydrazide ligands and the coordinated nitrate ions, Fig. 5 ▸. In addition, the non-coordinated nitrate anion is located in cavities along the *b* axis and serves as the acceptor site for six N—H⋯O hydrogen-bonding inter­actions (*i.e.* N1—H1*B*⋯O10, N2—H2⋯O12, N4—H4⋯O10, N4—H4⋯O11, N5—H5*A*⋯O10, and N5—H5*A*⋯O12) as shown in Fig. 6 ▸.

## Hirshfeld surface analysis

**Figure 7:**
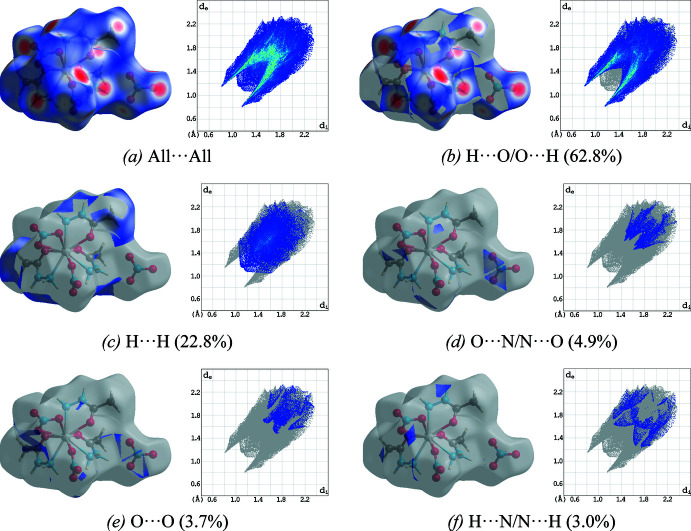
Two-dimensional fingerprint plots of **1**, showing (*a*) all inter­actions, and those delineated into (*b*) H⋯O/O⋯H, (*c*) H⋯H, (*d*) N⋯O/O⋯N, (*e*) O⋯O, and (*f*) H⋯N/N⋯H contacts [*d*_e_ and *d*_i_ represent the distances from a point on the Hirshfeld surface to the nearest atoms outside (external) and inside (inter­nal) the surface, respectively].

The Hirshfeld surface analysis (McKinnon *et al.*, 2007 ▸) and the associated two-dimensional fingerprint plot generation (Spackman & McKinnon, 2002 ▸) were carried out using *CrystalExplorer17* (Turner *et al.*, 2017 ▸) in order to qu­antify the nature of the inter­molecular inter­actions present in the crystal structure, and the results are shown in Figs. 7 ▸ and 8 ▸. The most significant contributions to the *d*_norm_ surfaces are H⋯O/O⋯H contacts (*i.e.* N—H⋯O hydrogen bonds), contributing 62.8% to the overall crystal packing of the title compound. The H⋯H contacts (representing van der Waals inter­actions) with a 22.8% contribution play a minor role in the stabilization of the crystal packing. All other N⋯O/O⋯N, O⋯O and H⋯N/N⋯H contacts make only negligible contributions to the Hirshfeld surface.

## Database survey

A ConQuest search of the Cambridge Structural Database (CSD, Version 5.42, September 2021 update; Bruno *et al.*, 2002 ▸; Groom *et al.*, 2016 ▸) for the structures of lanthanide complexes with acetohydrazide ligands gave ten hits, *viz*. Er [CECLEB (Pangani *et al.*, 1983 ▸), CECLEB10 (Agre *et al.*, 1984 ▸)], Dy [CECLIF (Pangani *et al.*, 1983 ▸), CECLIF10 (Pangani, Agre *et al.*, 1984 ▸)], Ho [CECLOL (Pangani *et al.*, 1983 ▸), CECLOL10 (Pangani, Agre *et al.*, 1984 ▸)], Pr (CUWFAB; Pangani, Machhoshvili *et al.*, 1984 ▸), Gd (FOYGIM; Brandão *et al.*, 2020 ▸), and Sm [ISNHSM (Zinner *et al.*, 1979 ▸), QITBIH (Theppitak *et al.*, 2018 ▸)]. In all of these complexes, the acetohydrazide ligand adopts a *μ*_2_-*κ* ^1^:*κ* ^1^ bidentate chelating coordination mode to bind the lanthanide(III) ion and the amine NH moiety of the acetohydrazide ligand can act as a donor site for inter­molecular hydrogen-bonding inter­actions, similar to that of the title compound.

## Synthesis and crystallization

A mixture of Tb(NO_3_)_3_·6H_2_O (45.3 mg, 0.1 mmol), acetohydrazide (14.8 mg, 0.2 mmol), and isopropyl alcohol (4 ml) was sealed in a 15 ml Teflon-lined steel autoclave and heated at 373 K for 24 h. The mixture was cooled to room temperature and colorless block-shaped crystals of the title compound (**1**) were obtained in 87% yield (39.3 mg, based on Tb^3+^ source). Analysis calculated (%) for C_6_H_18_N_9_O_12_Tb: C 12.71; H 3.20; N 22.23%. Found: C 12.44; H 3.96; N 21.89%.

## Refinement

Crystal data, data collection and structure refinement details are summarized in Table 2 ▸. All hydrogen atoms were located in difference-Fourier maps. All carbon-bound hydrogen atoms were placed in calculated positions and refined using a riding-model approximation with C—H = 0.96 Å and *U*_iso_(H) = 1.5*U*_eq_(C). All nitro­gen-bound hydrogen atoms were refined with a fixed distance N—H = 0.86 ± 0.02 Å.

## Supplementary Material

Crystal structure: contains datablock(s) I. DOI: 10.1107/S2056989022002298/yz2015sup1.cif

Supporting information file. DOI: 10.1107/S2056989022002298/yz2015Isup3.cdx

Structure factors: contains datablock(s) I. DOI: 10.1107/S2056989022002298/yz2015Isup4.hkl

CCDC reference: 2101422

Additional supporting information:  crystallographic information; 3D view; checkCIF report

## Figures and Tables

**Figure 8:**
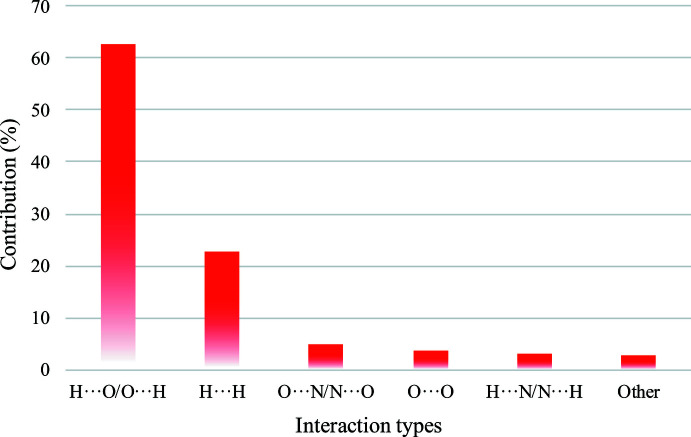
Qu­anti­tative results of different inter­molecular contacts contributing to the Hirshfeld surface of **1**.

**Table 1:** Hydrogen-bond geometry (Å, °)

*D*—H⋯*A*	*D*—H	H⋯*A*	*D*⋯*A*	*D*—H⋯*A*
N1—H1*A*⋯O8	0.84 (2)	2.37 (2)	2.950 (3)	126 (2)
N1—H1*B*⋯O10^i^	0.85 (2)	2.36 (2)	3.136 (3)	153 (3)
N2—H2⋯O11	0.85 (2)	2.69 (3)	3.070 (3)	109 (2)
N2—H2⋯O12	0.85 (2)	2.09 (2)	2.891 (2)	156 (3)
N3—H3*A*⋯O8	0.87 (2)	2.46 (3)	2.866 (3)	110 (2)
N3—H3*A*⋯O9^ii^	0.87 (2)	2.33 (2)	3.146 (3)	157 (2)
N3—H3*B*⋯O6^iii^	0.85 (2)	2.25 (2)	3.089 (3)	168 (3)
N4—H4⋯O10^iv^	0.87 (2)	2.34 (2)	3.102 (3)	147 (3)
N4—H4⋯O11^iv^	0.87 (2)	2.17 (2)	2.984 (3)	156 (3)
N5—H5*A*⋯O10^v^	0.86 (2)	2.58 (2)	3.176 (3)	128 (2)
N5—H5*A*⋯O12^v^	0.86 (2)	2.11 (2)	2.964 (2)	173 (3)
N5—H5*B*⋯O3^vi^	0.85 (2)	2.51 (2)	3.211 (2)	140 (2)
N6—H6⋯O7^vi^	0.85 (2)	2.17 (2)	2.999 (2)	166 (2)
N6—H6⋯O10^v^	0.85 (2)	2.74 (2)	3.170 (3)	114 (2)

Symmetry codes: (i) 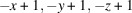 ; (ii) 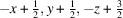 ; (iii) 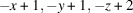 ; (iv) 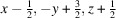 ; (v) 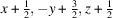 ; (vi) 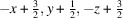 .

**Table 2:** Experimental details

Crystal data	
Chemical formula	[Tb(NO_3_)_2_(C_2_H_6_N_2_O)_3_]NO_3_
*M*_r_	567.21
Crystal system, space group	Monoclinic, *P*2_1_/*n*
Temperature (K)	296
*a*, *b*, *c* (Å)	10.9076 (3), 9.7786 (3), 16.8578 (5)
β (°)	90.791 (1)
*V* (Å^3^)	1797.90 (9)
*Z*	4
Radiation type	Mo *K*α
μ (mm^−1^)	4.02
Crystal size (mm)	0.28 × 0.21 × 0.2

Data collection	
Diffractometer	Bruker D8 QUEST CMOS
Absorption correction	Multi-scan (*SADABS*; Bruker, 2016 ▸)
*T*_min_, *T*_max_	0.471, 0.747
No. of measured, independent and observed [*I* > 2σ(*I*)] reflections	47511, 6876, 5752
*R*_int_	0.034
(sin θ/λ)_max_ (Å^−1^)	0.770

Refinement	
*R*[*F* ^2^ > 2σ(*F* ^2^)], *wR*(*F* ^2^), *S*	0.027, 0.044, 1.08
No. of reflections	6876
No. of parameters	293
No. of restraints	9
H-atom treatment	H atoms treated by a mixture of independent and constrained refinement
Δρ_max_, Δρ_min_ (e Å^−3^)	1.12, −1.13

Computer programs: *APEX3* and *SAINT* (Bruker, 2016 ▸), *SHELXT* (Sheldrick, 2015*a* ▸), *SHELXL* (Sheldrick, 2015*b* ▸), and *OLEX2* (Dolomanov *et al.*, 2009 ▸).

## supplementary crystallographic information

### Crystal data

**Table:**

[Tb(NO_3_)_2_(C_2_H_6_N_2_O)_3_]NO_3_	*F*(000) = 1112
*M**_r_* = 567.21	*D*_x_ = 2.096 Mg m^−^^3^
Monoclinic, *P*2_1_/*n*	Mo *K*α radiation, λ = 0.71073 Å
*a* = 10.9076 (3) Å	Cell parameters from 9937 reflections
*b* = 9.7786 (3) Å	θ = 3.0–33.1°
*c* = 16.8578 (5) Å	µ = 4.01 mm^−^^1^
β = 90.791 (1)°	*T* = 296 K
*V* = 1797.90 (9) Å^3^	Block, colourless
*Z* = 4	0.28 × 0.21 × 0.2 mm

### Data collection

**Table:**

Bruker D8 QUEST CMOS diffractometer	6876 independent reflections
Radiation source: sealed x-ray tube, Mo	5752 reflections with *I* > 2σ(*I*)
Graphite monochromator	*R*_int_ = 0.034
Detector resolution: 7.39 pixels mm^-1^	θ_max_ = 33.2°, θ_min_ = 2.8°
ω and φ scans	*h* = −16→14
Absorption correction: multi-scan (SADABS; Bruker, 2016)	*k* = −15→14
*T*_min_ = 0.471, *T*_max_ = 0.747	*l* = −25→25
47511 measured reflections	

### Refinement

**Table:**

Refinement on *F*^2^	Hydrogen site location: mixed
Least-squares matrix: full	H atoms treated by a mixture of independent and constrained refinement
*R*[*F*^2^ > 2σ(*F*^2^)] = 0.027	*w* = 1/[σ^2^(*F*_o_^2^) + (0.0127*P*)^2^ + 1.6017*P*] where *P* = (*F*_o_^2^ + 2*F*_c_^2^)/3
*wR*(*F*^2^) = 0.044	(Δ/σ)_max_ = 0.003
*S* = 1.08	Δρ_max_ = 1.12 e Å^−^^3^
6876 reflections	Δρ_min_ = −1.13 e Å^−^^3^
293 parameters	Extinction correction: SHELXL2018/3 (Sheldrick, 2015b), Fc^*^=kFc[1+0.001xFc^2^λ^3^/sin(2θ)]^-1/4^
9 restraints	Extinction coefficient: 0.00248 (11)

### Special details

**Table:**

Geometry. All esds (except the esd in the dihedral angle between two l.s. planes) are estimated using the full covariance matrix. The cell esds are taken into account individually in the estimation of esds in distances, angles and torsion angles; correlations between esds in cell parameters are only used when they are defined by crystal symmetry. An approximate (isotropic) treatment of cell esds is used for estimating esds involving l.s. planes.

### Fractional atomic coordinates and isotropic or equivalent isotropic displacement parameters (Å^2^)

**Table:**

	*x*	*y*	*z*	*U*_iso_*/*U*_eq_	
Tb1	0.53793 (2)	0.69267 (2)	0.81234 (2)	0.01794 (3)	
O1	0.53302 (15)	0.88900 (15)	0.73061 (9)	0.0316 (3)	
O2	0.48569 (13)	0.88947 (16)	0.88653 (10)	0.0310 (3)	
O3	0.68110 (13)	0.63211 (16)	0.71532 (9)	0.0275 (3)	
O4	0.5610 (2)	0.6421 (2)	0.95580 (11)	0.0535 (5)	
O5	0.68865 (16)	0.53978 (19)	0.88111 (12)	0.0425 (4)	
O6	0.7062 (2)	0.5199 (3)	1.00927 (15)	0.0780 (8)	
O7	0.47104 (13)	0.45766 (15)	0.79809 (10)	0.0297 (3)	
O8	0.27693 (15)	0.46317 (18)	0.76534 (12)	0.0430 (4)	
O9	0.37120 (17)	0.26983 (17)	0.77663 (12)	0.0431 (4)	
O10	0.51235 (17)	0.6218 (2)	0.38477 (11)	0.0446 (4)	
O11	0.57284 (17)	0.6504 (3)	0.50502 (13)	0.0598 (6)	
O12	0.38755 (14)	0.70400 (18)	0.47151 (9)	0.0337 (4)	
N1	0.4200 (2)	0.6697 (2)	0.67879 (12)	0.0302 (4)	
H1A	0.3464 (17)	0.647 (3)	0.6859 (16)	0.040 (8)*	
H1B	0.454 (3)	0.608 (3)	0.6519 (16)	0.050 (9)*	
N2	0.42249 (18)	0.7916 (2)	0.63406 (11)	0.0294 (4)	
H2	0.401 (3)	0.789 (3)	0.5854 (11)	0.048 (9)*	
N3	0.31650 (17)	0.7017 (2)	0.86115 (13)	0.0301 (4)	
H3A	0.265 (2)	0.694 (3)	0.8222 (13)	0.038 (7)*	
H3B	0.302 (3)	0.635 (2)	0.8919 (16)	0.051 (9)*	
N4	0.29181 (17)	0.8249 (2)	0.90180 (12)	0.0327 (4)	
H4	0.2186 (19)	0.842 (3)	0.9187 (18)	0.060 (10)*	
N5	0.74028 (16)	0.82302 (19)	0.82323 (11)	0.0243 (4)	
H5A	0.786 (2)	0.809 (3)	0.8643 (13)	0.040 (8)*	
H5B	0.722 (2)	0.9075 (18)	0.8197 (16)	0.040 (8)*	
N6	0.81190 (16)	0.79567 (19)	0.75554 (12)	0.0274 (4)	
H6	0.8761 (18)	0.842 (2)	0.7489 (15)	0.035 (7)*	
N7	0.6533 (2)	0.5651 (2)	0.95054 (14)	0.0433 (5)	
N8	0.36977 (17)	0.39549 (19)	0.77997 (11)	0.0290 (4)	
N9	0.49126 (17)	0.6579 (2)	0.45403 (11)	0.0286 (4)	
C1	0.48401 (19)	0.8957 (2)	0.66389 (13)	0.0258 (4)	
C2	0.4922 (3)	1.0227 (3)	0.61533 (16)	0.0426 (6)	
H2A	0.472625	1.100447	0.647550	0.064*	
H2B	0.435281	1.017233	0.571491	0.064*	
H2C	0.573927	1.032232	0.595665	0.064*	
C3	0.38049 (19)	0.9150 (2)	0.90919 (12)	0.0246 (4)	
C4	0.3478 (2)	1.0492 (3)	0.94566 (15)	0.0376 (5)	
H4A	0.401145	1.066733	0.990255	0.056*	
H4B	0.264351	1.046395	0.963032	0.056*	
H4C	0.356757	1.120640	0.907122	0.056*	
C5	0.77501 (18)	0.7031 (2)	0.70400 (13)	0.0246 (4)	
C6	0.8488 (2)	0.6862 (3)	0.63059 (16)	0.0421 (6)	
H6A	0.895336	0.603072	0.634062	0.063*	
H6B	0.903604	0.762411	0.625384	0.063*	
H6C	0.794817	0.682378	0.585182	0.063*	

### Atomic displacement parameters (Å^2^)

**Table:**

	*U*^11^	*U*^22^	*U*^33^	*U*^12^	*U*^13^	*U*^23^
Tb1	0.01473 (4)	0.01950 (5)	0.01956 (5)	0.00037 (4)	−0.00101 (3)	−0.00128 (4)
O1	0.0418 (9)	0.0248 (8)	0.0279 (8)	−0.0019 (7)	−0.0130 (7)	0.0027 (6)
O2	0.0230 (7)	0.0316 (8)	0.0386 (9)	−0.0015 (6)	0.0073 (6)	−0.0121 (7)
O3	0.0225 (7)	0.0280 (8)	0.0321 (8)	−0.0051 (6)	0.0061 (6)	−0.0090 (6)
O4	0.0599 (13)	0.0684 (13)	0.0320 (10)	0.0158 (11)	−0.0029 (9)	0.0067 (9)
O5	0.0319 (9)	0.0424 (10)	0.0530 (12)	0.0057 (8)	−0.0068 (8)	0.0097 (9)
O6	0.0641 (14)	0.102 (2)	0.0668 (15)	−0.0119 (14)	−0.0300 (12)	0.0547 (14)
O7	0.0236 (7)	0.0240 (7)	0.0414 (9)	−0.0037 (6)	−0.0017 (6)	−0.0023 (7)
O8	0.0224 (8)	0.0387 (10)	0.0679 (13)	0.0003 (7)	−0.0027 (8)	0.0016 (9)
O9	0.0467 (11)	0.0227 (8)	0.0598 (12)	−0.0082 (8)	−0.0065 (9)	0.0001 (8)
O10	0.0487 (11)	0.0525 (11)	0.0331 (9)	0.0088 (9)	0.0142 (8)	−0.0051 (8)
O11	0.0280 (9)	0.1001 (17)	0.0510 (12)	0.0141 (10)	−0.0115 (9)	−0.0132 (12)
O12	0.0214 (7)	0.0512 (10)	0.0284 (8)	0.0049 (7)	0.0009 (6)	−0.0029 (7)
N1	0.0331 (10)	0.0294 (10)	0.0278 (9)	−0.0056 (8)	−0.0048 (8)	−0.0015 (8)
N2	0.0332 (10)	0.0370 (11)	0.0178 (8)	−0.0010 (8)	−0.0041 (7)	0.0008 (8)
N3	0.0217 (8)	0.0310 (10)	0.0377 (11)	−0.0035 (8)	0.0021 (8)	−0.0029 (9)
N4	0.0198 (8)	0.0376 (11)	0.0410 (11)	0.0027 (8)	0.0094 (8)	−0.0069 (9)
N5	0.0234 (8)	0.0233 (9)	0.0261 (9)	−0.0011 (7)	−0.0032 (7)	−0.0024 (7)
N6	0.0213 (8)	0.0257 (9)	0.0352 (10)	−0.0075 (7)	0.0036 (7)	−0.0022 (8)
N7	0.0389 (11)	0.0437 (13)	0.0470 (13)	−0.0100 (10)	−0.0171 (10)	0.0220 (11)
N8	0.0277 (9)	0.0257 (9)	0.0337 (10)	−0.0065 (8)	0.0027 (8)	−0.0008 (8)
N9	0.0237 (9)	0.0321 (10)	0.0300 (9)	−0.0011 (7)	0.0041 (7)	0.0022 (8)
C1	0.0233 (10)	0.0299 (11)	0.0242 (9)	0.0054 (8)	0.0013 (8)	0.0019 (8)
C2	0.0429 (14)	0.0443 (14)	0.0404 (14)	−0.0028 (12)	−0.0072 (11)	0.0157 (12)
C3	0.0248 (9)	0.0301 (10)	0.0189 (9)	0.0048 (9)	0.0016 (7)	−0.0013 (8)
C4	0.0361 (12)	0.0364 (13)	0.0403 (13)	0.0112 (10)	0.0027 (10)	−0.0104 (11)
C5	0.0221 (9)	0.0221 (9)	0.0296 (10)	0.0016 (8)	0.0035 (8)	0.0010 (8)
C6	0.0414 (13)	0.0411 (14)	0.0445 (14)	−0.0059 (12)	0.0206 (11)	−0.0097 (12)

### Geometric parameters (Å, º)

**Table:**

Tb1—O1	2.3632 (15)	N2—H2	0.849 (17)
Tb1—O2	2.3690 (15)	N2—C1	1.316 (3)
Tb1—O3	2.3525 (14)	N3—H3A	0.865 (17)
Tb1—O4	2.4779 (19)	N3—H3B	0.850 (17)
Tb1—O5	2.4959 (17)	N3—N4	1.414 (3)
Tb1—O7	2.4220 (15)	N4—H4	0.867 (17)
Tb1—N1	2.587 (2)	N4—C3	1.313 (3)
Tb1—N3	2.5640 (19)	N5—H5A	0.857 (17)
Tb1—N5	2.5532 (18)	N5—H5B	0.851 (17)
O1—C1	1.240 (2)	N5—N6	1.417 (3)
O2—C3	1.240 (2)	N6—H6	0.845 (17)
O3—C5	1.254 (2)	N6—C5	1.314 (3)
O4—N7	1.261 (3)	C1—C2	1.491 (3)
O5—N7	1.262 (3)	C2—H2A	0.9600
O6—N7	1.222 (3)	C2—H2B	0.9600
O7—N8	1.294 (2)	C2—H2C	0.9600
O8—N8	1.232 (2)	C3—C4	1.494 (3)
O9—N8	1.230 (2)	C4—H4A	0.9600
O10—N9	1.244 (2)	C4—H4B	0.9600
O11—N9	1.231 (3)	C4—H4C	0.9600
O12—N9	1.257 (2)	C5—C6	1.494 (3)
N1—H1A	0.844 (17)	C6—H6A	0.9600
N1—H1B	0.845 (17)	C6—H6B	0.9600
N1—N2	1.411 (3)	C6—H6C	0.9600
			
O1—Tb1—O2	69.15 (6)	H3A—N3—H3B	106 (3)
O1—Tb1—O4	137.09 (7)	N4—N3—Tb1	111.81 (13)
O1—Tb1—O5	140.08 (6)	N4—N3—H3A	108.2 (18)
O1—Tb1—O7	135.12 (5)	N4—N3—H3B	109 (2)
O1—Tb1—N1	63.66 (6)	N3—N4—H4	120 (2)
O1—Tb1—N3	98.35 (6)	C3—N4—N3	118.22 (18)
O1—Tb1—N5	69.45 (6)	C3—N4—H4	121 (2)
O2—Tb1—O4	70.66 (7)	Tb1—N5—H5A	118.0 (19)
O2—Tb1—O5	113.77 (6)	Tb1—N5—H5B	106.4 (19)
O2—Tb1—O7	138.48 (5)	H5A—N5—H5B	110 (3)
O2—Tb1—N1	114.19 (6)	N6—N5—Tb1	109.48 (12)
O2—Tb1—N3	64.41 (6)	N6—N5—H5A	107.6 (19)
O2—Tb1—N5	76.73 (5)	N6—N5—H5B	104.8 (19)
O3—Tb1—O1	79.00 (6)	N5—N6—H6	118.1 (18)
O3—Tb1—O2	137.59 (5)	C5—N6—N5	119.68 (17)
O3—Tb1—O4	124.67 (6)	C5—N6—H6	122.1 (18)
O3—Tb1—O5	74.51 (6)	O4—N7—O5	115.89 (19)
O3—Tb1—O7	83.93 (5)	O6—N7—O4	121.8 (3)
O3—Tb1—N1	72.54 (6)	O6—N7—O5	122.3 (3)
O3—Tb1—N3	150.36 (6)	O8—N8—O7	119.45 (18)
O3—Tb1—N5	66.06 (5)	O9—N8—O7	117.96 (19)
O4—Tb1—O5	50.93 (7)	O9—N8—O8	122.57 (19)
O4—Tb1—N1	150.97 (7)	O10—N9—O12	120.03 (19)
O4—Tb1—N3	77.11 (7)	O11—N9—O10	119.8 (2)
O4—Tb1—N5	87.34 (7)	O11—N9—O12	120.1 (2)
O5—Tb1—N1	131.95 (7)	O1—C1—N2	121.1 (2)
O5—Tb1—N3	119.27 (7)	O1—C1—C2	120.9 (2)
O5—Tb1—N5	72.69 (6)	N2—C1—C2	118.0 (2)
O7—Tb1—O4	86.19 (7)	C1—C2—H2A	109.5
O7—Tb1—O5	70.93 (6)	C1—C2—H2B	109.5
O7—Tb1—N1	71.68 (6)	C1—C2—H2C	109.5
O7—Tb1—N3	77.33 (6)	H2A—C2—H2B	109.5
O7—Tb1—N5	137.71 (5)	H2A—C2—H2C	109.5
N3—Tb1—N1	79.81 (7)	H2B—C2—H2C	109.5
N5—Tb1—N1	121.66 (6)	O2—C3—N4	121.3 (2)
N5—Tb1—N3	140.98 (6)	O2—C3—C4	122.1 (2)
C1—O1—Tb1	125.30 (14)	N4—C3—C4	116.64 (19)
C3—O2—Tb1	123.84 (14)	C3—C4—H4A	109.5
C5—O3—Tb1	121.19 (13)	C3—C4—H4B	109.5
N7—O4—Tb1	96.95 (15)	C3—C4—H4C	109.5
N7—O5—Tb1	96.06 (14)	H4A—C4—H4B	109.5
N8—O7—Tb1	136.41 (13)	H4A—C4—H4C	109.5
Tb1—N1—H1A	111.2 (19)	H4B—C4—H4C	109.5
Tb1—N1—H1B	108 (2)	O3—C5—N6	121.60 (19)
H1A—N1—H1B	108 (3)	O3—C5—C6	121.0 (2)
N2—N1—Tb1	112.25 (13)	N6—C5—C6	117.39 (19)
N2—N1—H1A	109.1 (19)	C5—C6—H6A	109.5
N2—N1—H1B	108 (2)	C5—C6—H6B	109.5
N1—N2—H2	119 (2)	C5—C6—H6C	109.5
C1—N2—N1	117.60 (18)	H6A—C6—H6B	109.5
C1—N2—H2	122 (2)	H6A—C6—H6C	109.5
Tb1—N3—H3A	111.3 (19)	H6B—C6—H6C	109.5
Tb1—N3—H3B	110 (2)		
			
Tb1—O1—C1—N2	3.9 (3)	Tb1—O7—N8—O9	178.18 (15)
Tb1—O1—C1—C2	−176.58 (17)	Tb1—N1—N2—C1	1.4 (2)
Tb1—O2—C3—N4	8.5 (3)	Tb1—N3—N4—C3	0.0 (3)
Tb1—O2—C3—C4	−171.10 (16)	Tb1—N5—N6—C5	−6.9 (2)
Tb1—O3—C5—N6	14.8 (3)	N1—N2—C1—O1	−3.4 (3)
Tb1—O3—C5—C6	−164.56 (17)	N1—N2—C1—C2	177.1 (2)
Tb1—O4—N7—O5	−4.1 (2)	N3—N4—C3—O2	−5.3 (3)
Tb1—O4—N7—O6	174.7 (2)	N3—N4—C3—C4	174.4 (2)
Tb1—O5—N7—O4	4.0 (2)	N5—N6—C5—O3	−4.1 (3)
Tb1—O5—N7—O6	−174.7 (2)	N5—N6—C5—C6	175.3 (2)
Tb1—O7—N8—O8	−0.5 (3)		

### Hydrogen-bond geometry (Å, º)

**Table:**

*D*—H···*A*	*D*—H	H···*A*	*D*···*A*	*D*—H···*A*
N1—H1*A*···O8	0.84 (2)	2.37 (2)	2.950 (3)	126 (2)
N1—H1*B*···O10^i^	0.85 (2)	2.36 (2)	3.136 (3)	153 (3)
N2—H2···O11	0.85 (2)	2.69 (3)	3.070 (3)	109 (2)
N2—H2···O12	0.85 (2)	2.09 (2)	2.891 (2)	156 (3)
N3—H3*A*···O8	0.87 (2)	2.46 (3)	2.866 (3)	110 (2)
N3—H3*A*···O9^ii^	0.87 (2)	2.33 (2)	3.146 (3)	157 (2)
N3—H3*B*···O6^iii^	0.85 (2)	2.25 (2)	3.089 (3)	168 (3)
N4—H4···O10^iv^	0.87 (2)	2.34 (2)	3.102 (3)	147 (3)
N4—H4···O11^iv^	0.87 (2)	2.17 (2)	2.984 (3)	156 (3)
N5—H5*A*···O10^v^	0.86 (2)	2.58 (2)	3.176 (3)	128 (2)
N5—H5*A*···O12^v^	0.86 (2)	2.11 (2)	2.964 (2)	173 (3)
N5—H5*B*···O3^vi^	0.85 (2)	2.51 (2)	3.211 (2)	140 (2)
N6—H6···O7^vi^	0.85 (2)	2.17 (2)	2.999 (2)	166 (2)
N6—H6···O10^v^	0.85 (2)	2.74 (2)	3.170 (3)	114 (2)

Symmetry codes: (i) −*x*+1, −*y*+1, −*z*+1; (ii) −*x*+1/2, *y*+1/2, −*z*+3/2; (iii) −*x*+1, −*y*+1, −*z*+2; (iv) *x*−1/2, −*y*+3/2, *z*+1/2; (v) *x*+1/2, −*y*+3/2, *z*+1/2; (vi) −*x*+3/2, *y*+1/2, −*z*+3/2.
